# Clinical Characteristics of Young-Onset Versus Elderly-Onset Rheumatoid Arthritis: A Systematic Review and Meta-Analysis

**DOI:** 10.7759/cureus.74148

**Published:** 2024-11-21

**Authors:** Victor D Acuña-Rocha, Diego Regalado-Ceballos, Daniela A Salcedo-Soto, César A Ramos-Delgado, Jorge A Esquivel-Valerio, Ivan J Hernandez Galarza, Dionicio A Galarza-Delgado, Diana E Flores-Alvarado

**Affiliations:** 1 Internal Medicine, Hospital Universitario "Dr. José Eleuterio González", Monterrey, MEX; 2 Rheumatology, Plataforma INVEST Medicina UANL-Ker Unit Mayo Clinic México, Universidad Autonoma de Nuevo León, Monterrey, MEX; 3 Rheumatology, Hospital Universitario "Dr. José Eleuterio González", Monterrey, MEX

**Keywords:** aged, disease progression, elderly onset rheumatoid arthritis, rheumatoid arthritis, young adult

## Abstract

Rheumatoid arthritis (RA) is a chronic inflammatory disease with a prevalence of 1%, mainly affecting women aged 25-45. It is classified by the age of onset into young-onset rheumatoid arthritis (YORA, 16-65 years) and elderly-onset rheumatoid arthritis (EORA, over 65 years), with EORA often presenting suddenly with systemic symptoms and large joint involvement due to age-related immune changes.

This systematic review and meta-analysis compare the clinical and epidemiological characteristics of EORA and YORA. Observational studies were selected from PubMed, Scopus, Embase, Web of Science, and the Cochrane Central Database up to November 2023, focusing on a comparative analysis of both disease types with similar clinical progression and treatment duration limited to one month. Statistical analysis was performed in RStudio (Version 4.1.3, Posit Software, Boston, MA) using the "meta" package, applying a random effects model, inverse variance method, and Hartung-Knapp adjustment. Results for continuous variables were combined and grouped using the Cochrane formula, with medians and interquartile ranges transformed for uniformity. Four studies met the criteria. A trend was observed toward higher disease activity at diagnosis in the EORA group (mean difference (MD: 0.19, 95% CI -1.90 to 2.27), indicated by Disease Activity Score-28 (DAS28) and Simplified Disease Activity Index (SDAI) indices (MD 6.17, 95% CI -20.60 to 32.94). The EORA group also had higher Health Assessment Questionnaire (HAQ) scores (MD 0.21, 95% CI -0.03 to 0.46) and a greater number of painful (MD 1.31, 95% CI -0.86 to 3.47) and swollen joints (MD 2.35, 95% CI 0.77 to 3.92). Extra-articular manifestations, including rheumatoid nodules, lung involvement, and secondary Sjögren's syndrome, were more common in EORA patients (p < 0.004).

In conclusion, the findings suggest that patients with EORA present with more intense disease activity at onset, a higher prevalence of extra-articular manifestations, greater levels of disability, and more pronounced radiographic changes. Despite these initial differences, EORA patients ultimately achieve long-term remission rates similar to those with YORA.

## Introduction and background

Rheumatoid arthritis (RA) is a chronic inflammatory condition characterized by erosive arthritis and systemic organ involvement. It has a prevalence of 1% and can affect individuals of any age and sex, mainly young women between 25 and 45 years [1,2]. Recent studies have highlighted that elderly-onset rheumatoid arthritis (EORA) exhibits more severe disease activity and progression, along with worse clinical, functional, and radiographic outcomes when compared to young-onset rheumatoid arthritis (YORA). It can be classified into two subtypes based on the age of onset: YORA, which ranges from 16 to 65 years, and EORA after this age range, which represents a third of the entire population of RA patients [1-4].

The prevalence is higher in women; however, it changes with advancing age, with a greater proportion in men. A similar frequency is reached in men and women, as was observed in an Asian cohort [5]. With age, the immune system undergoes a process known as immunosenescence, where adaptive and innate immunity function incorrectly, and the ability to distinguish between the body's own and foreign bodies decreases [6-8]. Innate immunity is activated non-specifically, leading to chronic inflammation [8,9]. The immune system undergoes age-related changes, known as immunosenescence, which occurs prematurely in RA patients. Aging heightens nonspecific activity in the innate immune system, promoting chronic inflammation and comorbidities while impairing the adaptive immune system, disrupting tolerance, and increasing autoimmune disease risk [6,7]. 

T lymphocyte function in the adaptive immune response is most affected. It is characterized by thymic involution, oligoclonal peripheral T cell proliferation, telomere shortening, and loss of cluster of differentiation 28 (CD28) expression, which reduces the ability of T cells to recognize themselves and leads to an increase in autoimmune diseases, such as EORA, in the elderly [2,6-8,10-11]. Reports in the literature have found differences in the clinical presentation of EORA and YORA. The former has a more abrupt onset accompanied by general constitutional symptoms such as fatigue, weight loss, and more frequent involvement of large joints [12-15].

Some studies have found a greater degree of bone erosion and higher erythrocyte sedimentation rate (ESR) levels in EORA compared to YORA [16-18]. Regarding treatments, EORA and YORA differ. This finding is partly due to comorbidities, which occur more frequently in the former group. Moreover, there are currently no guidelines for the treatment of EORA, making most clinical decisions based on the YORA literature [6,19]. Patients with EORA also have a higher chance of developing adverse events. Patients with EORA are frequently treated with higher glucocorticoid doses and NSAIDs, and there is a trend toward using fewer disease-modifying antirheumatic drugs (DMARDs) or prescribing them at lower doses [3,5,18,20-25]. Although both groups benefit similarly from DMARD treatment, the risk of serious adverse events and a greater likelihood of switching to another type of treatment should be considered, especially in older patients [26-31].

Limited evidence exists regarding the clinical course and epidemiological characteristics of patients with EORA. To our knowledge, no systematic review has specifically focused on the differences between YORA and EORA in terms of clinical characteristics, prognosis, quality of life, mortality, morbidity, or systemic involvement. As a result, clinical decisions for patients with EORA are often based on evidence derived from their younger counterparts, potentially leading to suboptimal outcomes. Therefore, the objective of this study is to describe the clinical and epidemiological characteristics of EORA and YORA, the prevalence of comorbidities and extra-articular manifestations, and compare the serological data.

## Review

Materials and methods 

This study was developed with the Preferred Items for Systematic Review Protocols and Meta-Analyses (PRISMA) statement and was registered in the International Prospective Register of Systematic Reviews (PROSPERO; registration number CRD42021238906). A systematic literature search was conducted on October 16, 2023, in the databases Embase (1974-October 2023), Scopus, Medline, and Web of Science (1946-October 2023). The search strategy included a combination of medical subject headings (MeSH): "arthritis", "rheumatoid", "elderly", "young adult", along with keywords relevant to the study population ("rheumatoid arthritis"), the intervention ("elderly", "advanced age", "older", "elderly", "aging", "late onset"), and the comparison group ("young", "onset in youth"), with language restrictions to English and Spanish. Observational studies that evaluated any outcome or characteristic comparing EORA and YORA were included. Clinical progression had to be one month or less after starting treatment with a comparable duration from the onset of symptoms. The search was limited to studies in English and Spanish. Studies that did not compare EORA and YORA characteristics were excluded, ensuring that the review focused on directly comparing disease manifestations and progression across age groups.

Selection Process 

Two independent research team members (VDAR and DRC) worked in duplicate to collect information from the included studies. The selection process was divided into a title/abstract phase and a full-text phase. A pilot test was conducted among reviewers before each phase to ensure adequate rater agreement (a kappa index equal to or greater than 0.7). This phase was followed by a data extraction phase, in which the studies were analyzed qualitatively and quantitatively. Data collection conflicts were resolved by consensus and with the intervention of a third reviewer (DEFA).

Study Reliability 

The risk of bias was assessed using the Newcastle-Ottawa Scale (NOS) [32], which evaluates three key domains: selection of study participants, comparability of study groups, and ascertainment of outcomes. A pair of reviewers (VDAR and DEFA) independently assessed each included study to ensure objectivity and minimize bias. Discrepancies between the reviewers were resolved through a joint review and consensus process, which involved a detailed re-examination of the study criteria in question. In cases where consensus was not reached, a third reviewer (if applicable) was consulted for arbitration.

Statistical Analysis

The meta-analysis was performed in RStudio (Version 4.1.3, Posit Software, Boston, MA) with the "meta" package, using a random effects model, the inverse variance method, and Hartung-Knapp adjustment. Continuous variables were expressed as means and standard deviations and were analyzed using the difference in means. The Cochrane formula was used to combine groups and group the results of continuous variables of the YORA and EORA subgroups. For variables reported as medians and interquartile ranges, a transformation to means and standard deviations was performed with the quantile estimation method of McGrath [33]. 

Results 

Study Selection and Characteristics 

**Figure 1:**
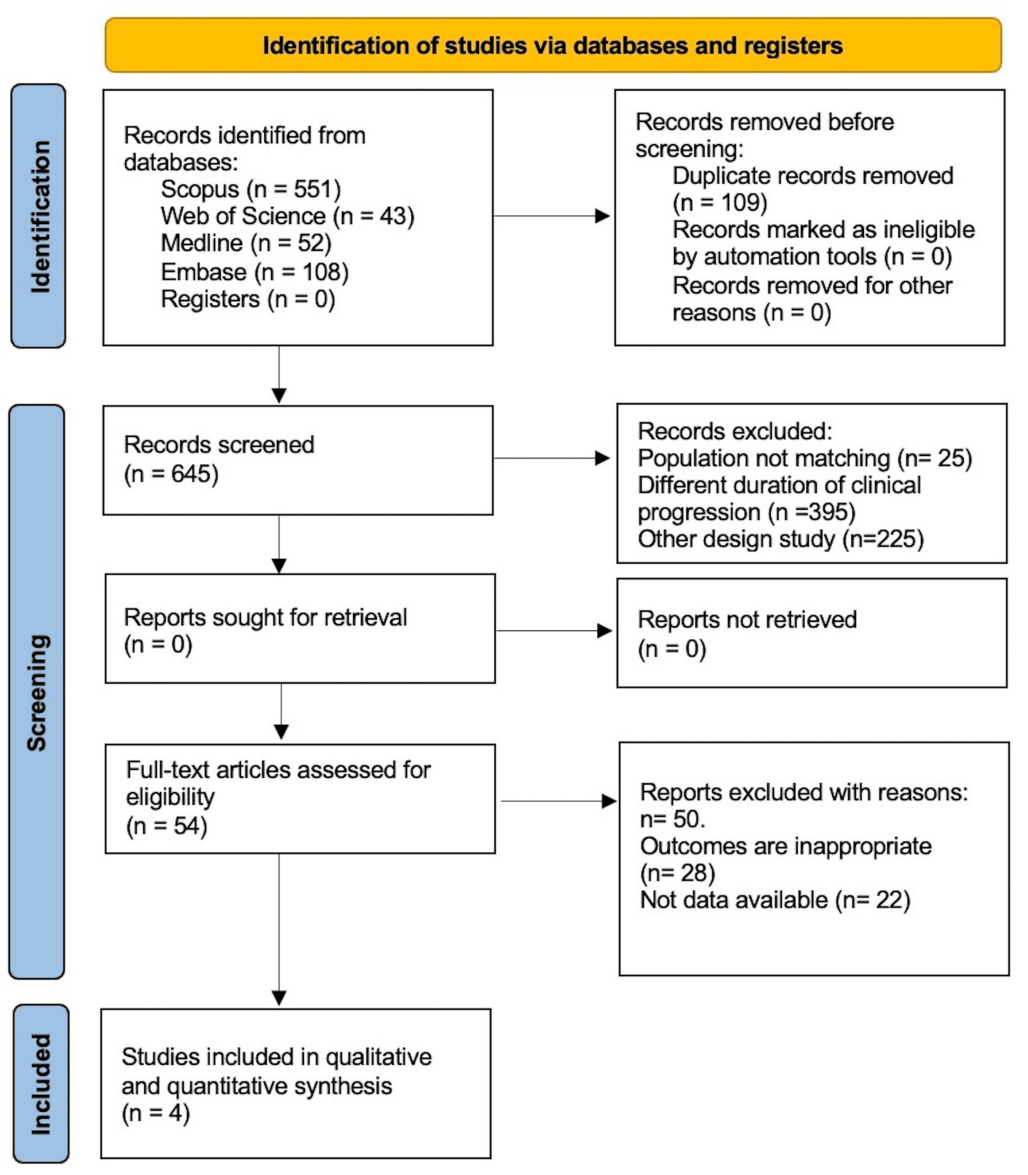
Systematic flow diagram Preferred Items for Systematic Review Protocols and Meta-Analyses (PRISMA) flow chart on selection and inclusion of studies.

The systematic search identified 645 studies, of which 54 progressed to the full-text review phase. Following this review, four studies met all the inclusion criteria and were selected for data extraction, which involved qualitative and quantitative analyses (Figure 1). All the included studies defined 60 years of age as the cut-off point to classify patients into the EORA and YORA groups [18,34-36]. Regarding disease duration, three studies were within a range of four to six months [18,34,35], in contrast to a study in Malaysia [36], where the average was 100 months (Table 1).

**Table 1:** Main characteristics of the included studies ACPA: anti-citrullinated protein antibodies; CRP: C-reactive protein; DAS28-ESR: Disease Activity Score-28 Based on Erythrocyte Sedimentation Rate; EORA: elderly-onset rheumatoid arthritis; ESR: erythrocyte sedimentation rate; HAQ: Health Assessment Questionnaire; NA: not available; SD: standard deviation; SDAI: Simplified Disease Activity Index; YORA: young-onset rheumatoid arthritis

First author, year	Country	Study type	Population		Age group	Mean age (SD)	Total sample size	Duration of clinical evolution (months)	DAS28-ESR (SD)	SDAI (SD)	HAQ (SD)	VSG (mm/h) (SD)	PCR (mg/dL) (SD)	Rheumatoid factor (%)	ACPA (U/mL) (SD)
Murata et al., 2019 [18]	Japan	Cohort	EORA	≥60		69.6 (6.9)	122	5.3 (4.1)	5 (1.5)	23.4 (15.2)	0.7 (0.7)	50.8 (27.8)	3.08 (4.4)	78 (63.9)	276.3 (328.7)
			YORA	<60		47 (8.9)	117	6.1 (3.9)	4 (1.2)	15 (10)	0.4 (0.4)	28.9 (21.7)	1.2 (2.6)	85 (72.6)	178.7 (173.5)
Krams et al., 2016 [34]	France	Cohort	EORA	≥60		64.6 (2.72)	118	4.9 (3.19)	NA	32.15 (13.11)	1.06 (0.86)	25 (28.15)	13 (28.15)	56 (47)	NA
			YORA	<60		45.85 (10.93)	580	4.92 (3.36)	NA	27.97 (12.93)	0.94 (0.74)	21.54 (17.31)	8.68 (15.16)	316 (54.4)	NA
Romão et al., 2020 [35]	England	Cohort	EORA	≥60		71 (8)	41	5.7 (3.5)	5.8 (1.4)	NA	1.6 (0.8)	42.4 (28.1)	2.3 (3.3)	NA	NA
			YORA	<60		44 (11)	99	6 (3.2)	5.6 (1.4)	NA	1.4 (0.7)	33.8 (28.6)	1.5 (2.3)	NA	NA
Rajalingham et al., 2021 [36]	Malaysia	Cohort	EORA	≥60		67.19 (2.49)	69	96.72 (60.48)	3.29 (1.37)	NA	1.47 (0.87)	54.20 (20.50)	1.33 (1.37)	47 (68.11)	84.52 (117.97)
			YORA	<60		31.93 (5.10)	82	104.4 (43.56)	3.96 (1.68)	NA	1.58 (0.71)	67.04 (23.60)	3.79 (8.11)	62 (75.61)	151.14 (138.02)

Regarding disease activity scales, the studies by Romão et al. and Rajalingham et al. [35,36] did not report the Simplified Disease Activity Index (SDAI). Krams et al. did not include the Disease Activity Score-28 Based on Erythrocyte Sedimentation Rate (DAS28-ESR) scale [34]. The average age in the EORA group was 68.09 years, while in the YORA group, it was 42.19 years. Regarding anti-citrullinated protein antibodies (ACPA), Murata et al. [18] reported levels of 276.3 U/mL (328.7) in EORA and 178.7 U/mL (173.5) in YORA. Likewise, Rajalingham et al. [36] found levels of 84.52 U/mL (117.97) in EORA and 151.14 U/mL (138.02) in YORA.

In a study conducted by Krams et al. [34], 34% of EORA and 47.41% of YORA patients tested positive for these antibodies. Romão et al. [35] reported the presence of rheumatoid factor (RF) and ACPA, indicating that 61% of patients with EORA and 69% with YORA were positive for both markers.

As for treatment in all studies included in the systematic review [18,34-36], there was a reduction in the use of DMARDs and biologicals and an increase in the use of steroids in the EORA group, as opposed to the YORA group, which showed greater use of DMARDs, biologicals and less steroid use. This trend continued in the follow-up of the included prospective studies [18,34,35]. With regard to comorbid conditions, analyses of three cohort studies elucidated that patients with EORA present a spectrum of comorbidities, including diabetes mellitus (DM), high blood pressure (HTN), osteoarthritis (OA), chronic kidney disease (CKD), osteoporosis, and ischemic heart disease [18,34,35]. On the other hand, patients with EORA more frequently presented extra-articular manifestations, specifically rheumatoid nodules, interstitial lung disease (ILD), pleural effusion, primary Sjögren's syndrome, and cutaneous vasculitis.

Meta-Analysis of Disease Activity, Joint Involvement, Quality of Life, Acute Phase Reactants, and RF 

**Figure 2:**
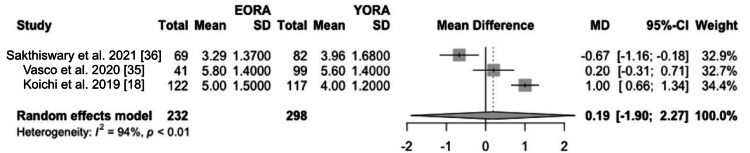
DAS28-ESR Forest plot showing mean differences in DAS28-ESR scores between EORA and YORA patients. DAS28-ESR: Disease Activity Score-28 Based on Erythrocyte Sedimentation Rate; EORA: elderly-onset rheumatoid arthritis; MD: mean difference; YORA: young-onset rheumatoid arthritis

**Figure 3:**
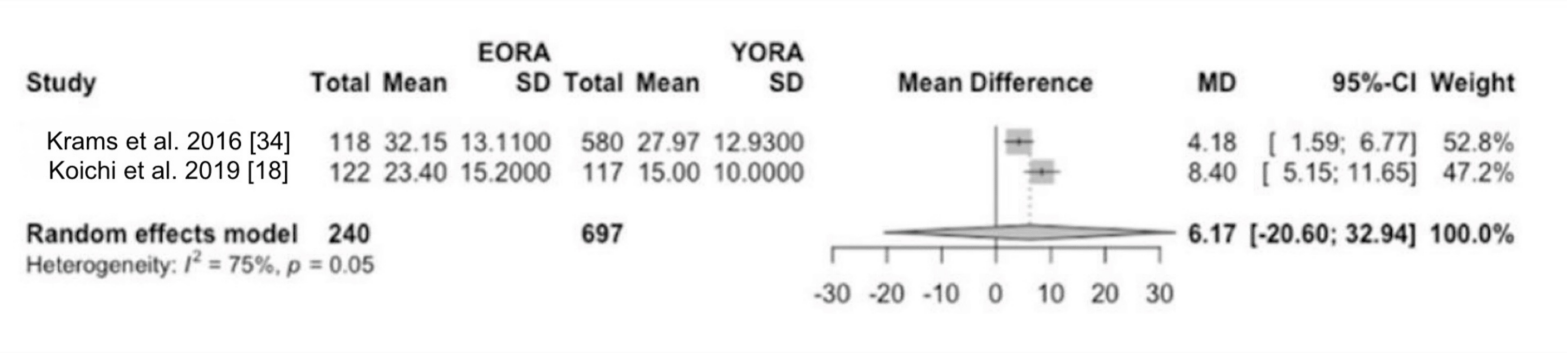
SDAI Forest plot showing mean differences in SDAI scores between EORA and YORA patients. EORA: elderly-onset rheumatoid arthritis; MD: mean difference; SDAI: Simplified Disease Activity Index; YORA: young-onset rheumatoid arthritis

**Figure 4:**
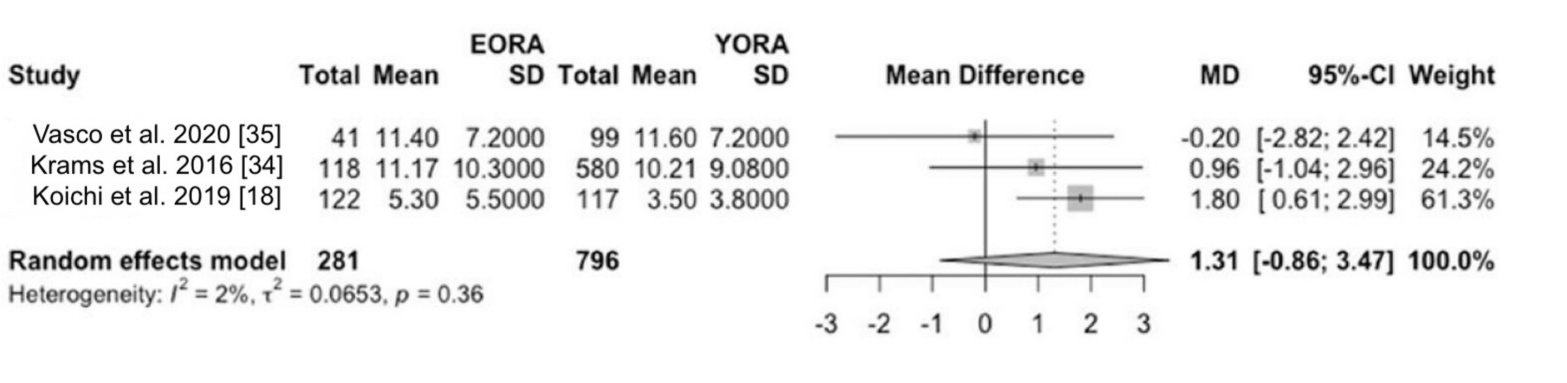
Tender and painful joints Forest plot showing mean differences in scores for tender and painful joints between EORA and YORA patients. EORA: elderly-onset rheumatoid arthritis; MD: mean difference; YORA: young-onset rheumatoid arthritis

**Figure 5:**
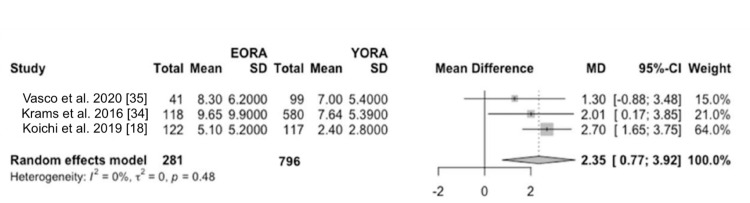
Swollen joints Forest plot showing mean differences in scores for swollen joints between EORA and YORA patients. EORA: elderly-onset rheumatoid arthritis; MD: mean difference; YORA: young-onset rheumatoid arthritis

Concerning disease activity at diagnosis, patients classified as EORA showed significantly higher disease activity than YORA patients. This finding was quantified using a mean difference (MD) of 0.19 (95% CI -1.90 to 2.27) based on the DAS28 index (Figure 2) and an MD of 6.17 (95% CI -20.60 to 32.94) using the SDAI (Figure 3) [8,34-36]. However, this initial difference in disease activity decreased after one and two years of treatment, indicating comparable long-term therapeutic efficacy between both groups in three of our studies [18,34,35]. Additionally, in terms of measurements of painful joints (MD 1.31, 95% CI -0.86 to 3.47) (Figure 4) and swollen joints (MD 2.35, 95% CI 0.77 to 3.92) (Figure 5), patients with EORA had a higher prevalence of painful (p < 0.01) and swollen (p < 0.001) joints in three of our included studies.

**Figure 6:**
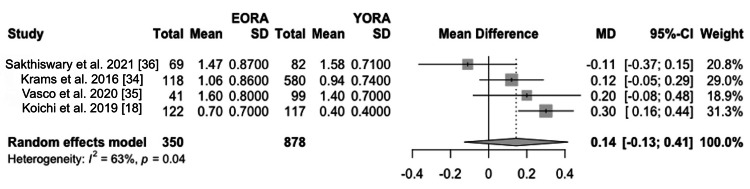
HAQ Forest plot showing mean differences in HAQ scores between EORA and YORA patients. EORA: elderly-onset rheumatoid arthritis; HAQ: Health Assessment Questionnaire; MD: mean difference; YORA: young-onset rheumatoid arthritis

**Figure 7:**
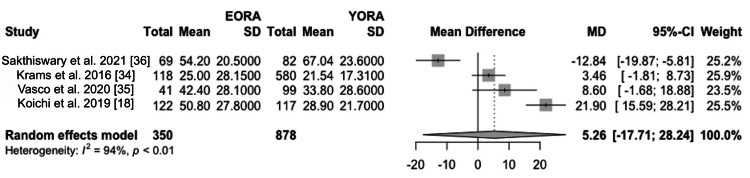
ESR Forest plot showing mean differences in ESR scores between EORA and YORA patients. EORA: elderly-onset rheumatoid arthritis; ESR: erythrocyte sedimentation rate; MD: mean difference; YORA: young-onset rheumatoid arthritis

**Figure 8:**
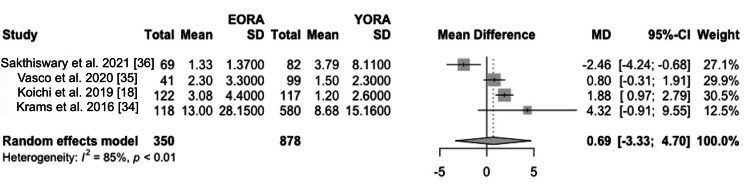
CRP Forest plot showing mean differences in CRP scores between EORA and YORA patients. CRP: C-reactive protein; EORA: elderly-onset rheumatoid arthritis; MD: mean difference; YORA: young-onset rheumatoid arthritis

**Figure 9:**
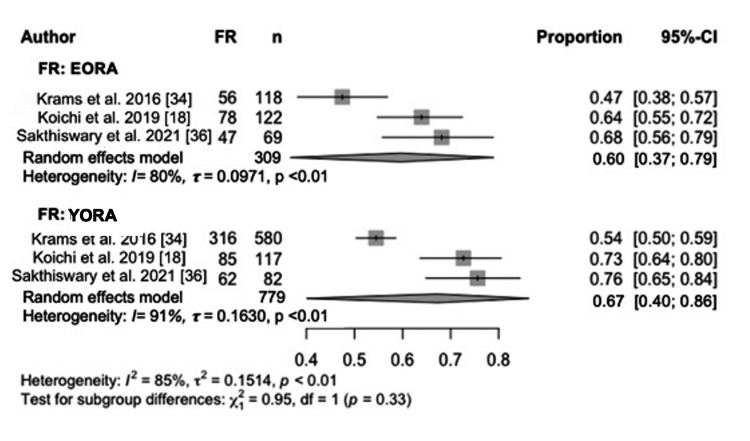
RF Forest plot showing the proportion of RF positivity in EORA and YORA patients. EORA: elderly-onset rheumatoid arthritis; MD: mean difference; RF: rheumatoid factor; YORA: young-onset rheumatoid arthritis

However, this difference leveled off after two years of treatment [18,34,35]. In terms of symptoms and their prevalence at the time of diagnosis, in terms of general health assessment, analyses revealed that the EORA group had higher scores on the Health Assessment Questionnaire (HAQ) (MD 0.14, 95% CI -0.13 to 0.41) [18,34-36] (Figure 6). At the first visit, an elevated ESR (Figure 7) and C-reactive protein (CRP) (Figure 8) were found in the EORA group (MD 5.26, 95% CI -17.71 to 28.24 for ESR; MD 0.69, 95% CI -3.33 to 4.70 for PCR) [18,34-36]. Considering serological markers, RF was found in 67% of YORA patients and 60% of EORA patients, highlighting the differences in serological profiles between the groups [18,34,36] (Figure 9). In one study, extra-articular manifestations, such as rheumatoid nodules, pulmonary involvement, and secondary Sjögren's syndrome, were more common in the EORA group (p < 0.004) [35].

Radiographic progression was greater in patients with EORA [18,34-36] evaluated by the Larsen score [18], modified total sharp score (mTSS) indices [34], Sharp-van der Heijde score (SvdH) score [35], and the modified sharp score (MSS) score [36]. In one study, this greater progression was significantly associated with EORA (OR 5.6; p < 0.001) and the presence of positive ACPA (OR 4.0; p < 0.001) [18]. Finally, concerning treatment tolerance and response, even though the therapeutic regimen adhered to international guidelines in all the cohorts studied, patients with EORA exhibited reduced tolerance to methotrexate after one year. This resulted in the necessity to administer lower doses due to the presence of associated comorbidities [18,34,35].

Risk of Bias

The risk of bias was assessed using the NOS [32], which evaluates studies across three key stages: selection, comparability, and outcome. The assessment showed that all studies had representative cohorts with clearly defined inclusion and exclusion criteria, and patients were consistently drawn from the same geographic area. The required number of patients was met in all studies, and robust protocols for patient registration were followed [18,34-36].

In terms of comparability, all studies controlled appropriately for confounding variables such as sex, age, and duration of disease symptoms through matching or statistical adjustments [18,34-36]. Regarding outcomes, all cohort studies had follow-up periods of at least one year, ensuring sufficient observation time [18,34,35]. Follow-up was complete, and outcomes were assessed using validated and objective measures. Overall, the NOS evaluation demonstrated high methodological quality and minimal risk of bias in the included studies.

Discussion

In our systematic review of four studies [18,34-36], we analyzed data from 1,228 patients and explored the impact of age of disease onset on RA progression and treatment response. Patients with EORA had higher disease activity at diagnosis, evidenced by higher DASS28-ESR and SDAI scores, although this was not statistically significant in the meta-analysis. However, a trend toward greater disease activity in EORA was consistent with other studies [5,37,38], which found non-significant differences but suggested a trend toward greater activity in EORA. Furthermore, clinically, all patients in those studies fell within the same severity range with similar clinical impact.

Longitudinal follow-up of patients with EORA and YORA during treatment intervals of one and two years demonstrated that, despite initially having greater severity in EORA, the probability of achieving remission or maintaining low disease activity was similar to that observed in the YORA group [18,34-36], which is consistent with previous research [37]. Other studies indicated that the EORA group experienced a higher remission rate, possibly explained by the higher proportion of seronegative patients within the EORA population.

This finding could be attributed to immunosenescence in EORA, where similar remission rates are achieved even with less aggressive treatments than those in YORA cohorts. This hypothesis deserves further exploration through rigorous clinical trials.

Our analysis indicated that the EORA group had higher HAQ scores (MD 0.14, 95% CI -0.13 to 0.41), reflecting findings from other studies [3,5,17,39,40-42] where a higher HAQ score was observed in patients with EORA. The higher HAQ scores in EORA patients may reflect a combination of factors such as increased physical frailty, comorbidities associated with aging, and baseline functional decline, which are less common in younger patients with YORA. Differences in the magnitude of HAQ scores between studies may also be attributable to variations in HAQ measurement methods, differences in study populations (e.g., geographic, ethnic, or socioeconomic contexts), or statistical adjustments for confounding factors such as comorbidities.

In this review, we found that there are higher initial levels of ESR and CRP in patients with EORA [18,34-36], aligning with other studies [3,5,17,39,41,43] and contrasting with others [38,40,43]. These discrepancies could potentially be explained by differences in the duration of disease, as the studies showing lower levels included patients with a disease duration of at least four years, during which inflammation may have been moderated by prolonged treatment, clinical remission, or advanced structural damage altering inflammatory responses. Additionally, the higher ESR and CRP levels in EORA may reflect an amplified inflammatory response due to immunosenescence, a characteristic of aging immune systems. Early access to effective therapies such as biologics or DMARDs in certain cohorts could also influence these findings by reducing inflammatory markers.

Furthermore, higher baseline levels of RF were observed in all of our YORA patients [18,34-36], which is consistent with other investigations [6,17,38,41,43,44]. This may be due to a higher proportion of seropositive patients in YORA cohorts, reflecting a closer association between RF seropositivity and younger onset disease. Variability in RF levels between studies may also stem from differences in the inclusion criteria, serological testing methods, and patient characteristics, such as genetic predispositions or environmental factors that influence RF production.

Regarding comorbidities, patients with EORA frequently showed DM, HTN, OA, CKD, osteoporosis, and ischemic heart disease [18,35,36], similar to other studies [3,5,38,39]. Regarding extra-articular manifestations, one of our studies [35] reported a higher prevalence in EORA, such as rheumatoid nodules, ILD, pleural effusion, primary Sjögren's syndrome, and cutaneous vasculitis, contrasting with other studies [5,39] where the YORA group showed more ILD involvement, possibly due to a longer disease course in the study.

Murata et al. reported higher ACPA titers in EORA than in the YORA group of Rajalingham et al. [18,36], which had the highest titles. A longer disease duration in the Rajalingham study could explain the lower ACPA levels in EORA, which is compatible with the results of other studies [17,37,38,40] and contrasts with another [3], where the YORA group had a higher prevalence of the protein tyrosine phosphatase non-receptor type 22 (PTPN22) T polymorphism associated with higher ACPA titers.

Regarding radiographic progression, patients with EORA showed more severe involvement in our cohort studies [18,34,35], which is consistent with other studies [3,17,43]. On the other hand, one study [36] showed greater radiographic involvement in YORA. Findings from other studies [5,43] suggest this could be due to longer disease progression.

Our systematic review found a higher prevalence of swollen, painful, and tender joints at diagnosis in EORA, unlike other studies [3,17,37]. However, one study [18] reported increased activity at diagnosis with similar activity at two years. The poor tolerance to methotrexate observed in patients with EORA after one year of treatment [18,34,35] emphasizes the importance of individualizing patient treatment considering concomitant conditions. It suggests future research on treatment doses in different age groups to optimize therapeutic outcomes.

## Conclusions

This systematic review and meta-analysis show that patients with EORA tend to present with higher disease activity at diagnosis, including elevated DASS28-ESR and SDAI scores, more severe radiographic changes, and higher levels of inflammatory markers, though these differences were not statistically significant. Despite the initial severity and a higher prevalence of comorbidities and extra-articular manifestations, EORA patients achieve a similar long-term remission rate to YORA patients, likely due to age-related immune changes such as immunosenescence, which may allow for effective disease control with potentially less aggressive treatment regimens.

These findings underscore the need for personalized treatment in EORA, emphasizing the importance of considering the unique clinical profiles of older RA patients to optimize therapeutic outcomes and improve long-term management.
